# Sleep-Disordered Breathing Is Associated With Reduced Left Atrial Strain Measured by Cardiac Magnetic Resonance Imaging in Patients After Acute Myocardial Infarction

**DOI:** 10.3389/fmed.2022.759361

**Published:** 2022-02-16

**Authors:** Michael Wester, Jan Pec, Simon Lebek, Christoph Fisser, Kurt Debl, Okka Hamer, Florian Poschenrieder, Stefan Buchner, Lars S. Maier, Michael Arzt, Stefan Wagner

**Affiliations:** ^1^University Heart Center Regensburg, University Hospital Regensburg, Regensburg, Germany; ^2^Department of Radiology, University Hospital Regensburg, Regensburg, Germany; ^3^Department of Internal Medicine, Cham Hospital, Cham, Germany

**Keywords:** AHI, atrial strain, left atrium, cardiac magnetic resonance imaging, CMR, sleep-disordered breathing

## Abstract

**Aims:**

Sleep disordered breathing (SDB) is known to cause left atrial (LA) remodeling. However, the relationship between SDB severity and LA dysfunction is insufficiently understood and may be elucidated by detailed feature tracking (FT) strain analysis of cardiac magnetic resonance images (CMR). After myocardial infarction (MI), both the left ventricle and atrium are subjected to increased stress which may be substantially worsened by concomitant SDB that could impair consequential healing. We therefore analyzed atrial strain in patients at the time of acute MI and 3 months after.

**Methods and Results:**

40 patients with acute MI underwent CMR and polysomnography (PSG) within 3–5 days after MI. Follow-up was performed 3 months after acute MI. CMR cine data were analyzed using a dedicated FT software. Atrial strain (ε) and strain rate (SR) for atrial reservoir ([ε_s_]; [SR_s_]), conduit ([ε_e_]; [SR_e_]) and booster function ([ε_a_]; [SR_a_]) were measured in two long-axis views. SDB was defined by an apnea-hypopnea-index (AHI) ≥15/h. Interestingly, LA ε_s_ and ε_e_ were significantly reduced in patients with SDB and correlated negative with AHI as a measure of SDB severity at both baseline and follow-up. Intriguingly, patients that exhibited a reduced AHI at follow-up were more likely to have developed improved atrial reservoir and conduit strain (linear regression, *p*=0.08 for ε_s_ and ε_e_). Patients with improved SDB (ΔAHI < −5/h) exhibited a mean improvement of LA reservoir strain of +7.2 ± 8.4% whereas patients with SDB deterioration (ΔAHI> + 5/h) showed a mean decrease of −5.3 ± 11.0% (*p* = 0.0131). Similarly, the difference for LA conduit function was +4.8 ± 5.9% (ΔAHI < −5/h) vs −3.6 ± 8.8% (ΔAHI> +5/h). Importantly, conventional volumetric parameters for atrial function (LA area, LA volume index) did not correlate with AHI at baseline or follow-up.

**Conclusion:**

Our results show that LA function measured by CMR strain but not by volumetry is impaired in patients with SDB during acute cardiac injury. Consistent with a mechanistic association, improvement of SBD at follow-up resulted in improved LA strain. LA strain measurement might thus provide insight into atrial function in patients with SDB.

## Introduction

Sleep disordered breathing (SDB) is a highly prevalent disease, especially in patients with heart failure (1, 2). SDB and especially obstructive sleep apnea negatively affect the heart through several closely interconnected mechanisms. Breathing against the obstructed pharynx leads to stark negative intrathoracic pressures with increased cardiac wall stress and increased venous return and subsequent right ventricular distension (3). In combination with hypoxia, this leads to stimulation of the sympathetic nervous system which elevates blood pressure, increases myocardial oxygen demand, and favors arrhythmias (2). Intermittent hypoxia increases the production of myocardial reactive oxygen species which can impair myocardial and endothelial function (2). Also, hypoxia stimulates the formation of myocardial fibrosis by activation of angiotensin II and aldosterone (2). These detrimental mechanisms can cause both acute (2, 4, 5) and chronic heart failure (2, 6, 7) and consequentially, SDB is a predictor of worse cardiovascular outcome in patients with cardiovascular disease (2). Our group has demonstrated that SDB impairs myocardial salvage in patients with acute myocardial infarction (MI) leading to significantly larger scar tissue areas and altered ventricular remodeling (8, 9).

Research is often focused on cardiac ventricular function, however, emerging evidence indicates that LA function plays an important role in cardiovascular performance and that LA dysfunction is associated with adverse cardiovascular outcome such as a higher risk for cardiovascular events (10), new onset of atrial fibrillation (11), stroke (12), reduced exercise tolerance in heart failure (13), and higher mortality after MI (14). SDB does not only affect the ventricles but also the atria. Due to their thinner and less muscular walls, the atria are more susceptible to the intrathoracic pressure changes in SDB resulting in LA enlargement (LA area and LA volume index) (15, 16). Due to methodological shortcomings, the direct volumetric evaluation of atrial function (i.e., ejection fraction) is currently not recommended for routine diagnostics (17). In contrast, analysis of myocardial deformation by feature tracking strain analysis provides a tool for the evaluation of atrial function. Echocardiographic strain analysis has revealed that LA function is reduced in patients with SDB compared to healthy individuals (18, 19). However, echocardiographic evaluation of atrial function is often hampered as it is difficult to capture the complete atria and atrial walls in many patients due to often insufficient acoustic windows. Additionally, as the atria are the farthest chambers from the ultrasonic probe, technical limitations may further reduce the usefulness of echocardiography for the quantification of atrial function. In contrast, cardiac anatomy – especially of the atria – can be accurately visualized in cardiac magnetic resonance imaging (CMR). Feature tracking analysis can be applied to CMR cine data and atrial strain can be derived (20, 21). This method has recently been successfully used for the characterization of LA function in healthy individuals (22) and in patients with acute myocarditis (23), heart failure with preserved ejection fraction (13) or tako-tsubo syndrome (24). Impaired LA function was associated with morbidty and mortality in patients with tako-tsubo syndrome (24), in the general population (25), and in patients with heart failure (25). On a mechanistical level, reduced LA strain is associated with histologically assessed fibrofatty remodeling (26).

However, currently there is no data on atrial strain and the influence of SDB during acute myocardial ischemia. We therefore investigated the influence of SDB on atrial strain by CMR in patients presenting with acute MI and at 3-month follow up. Interestingly, at each time point atrial function was decreased in patients with SDB. However, the change in SDB severity from baseline to follow-up correlated with the change in atrial strain function, suggesting a close causal connection between SDB severity and atrial function.

## Methods

We performed a sub-analysis of a prospective observational study in patients with acute MI that were enrolled at University Medical Center Regensburg (Regensburg, Germany) between March 2009 and March 2012. Details of the study design have been published previously (27).

Key inclusion criteria for this sub-analysis were as follows: patients (age 18–80 years) with a first acute MI and PCI treated at the University Hospital Regensburg within 24 h after symptom onset were eligible for inclusion. Exclusion criteria were previous MI or previous PCI, indication for surgical myocardial revascularization, cardiogenic shock, contraindications for CMR, and severe comorbidities (e.g., lung disease, stroke, treated SDB). The study protocol was reviewed and approved by the local institutional ethics committee (Regensburg, 08–151) and is in accordance with the Declaration of Helsinki and Good Clinical Practice. A written informed consent was obtained from all patients prior to enrolment.

**Figure 1:**
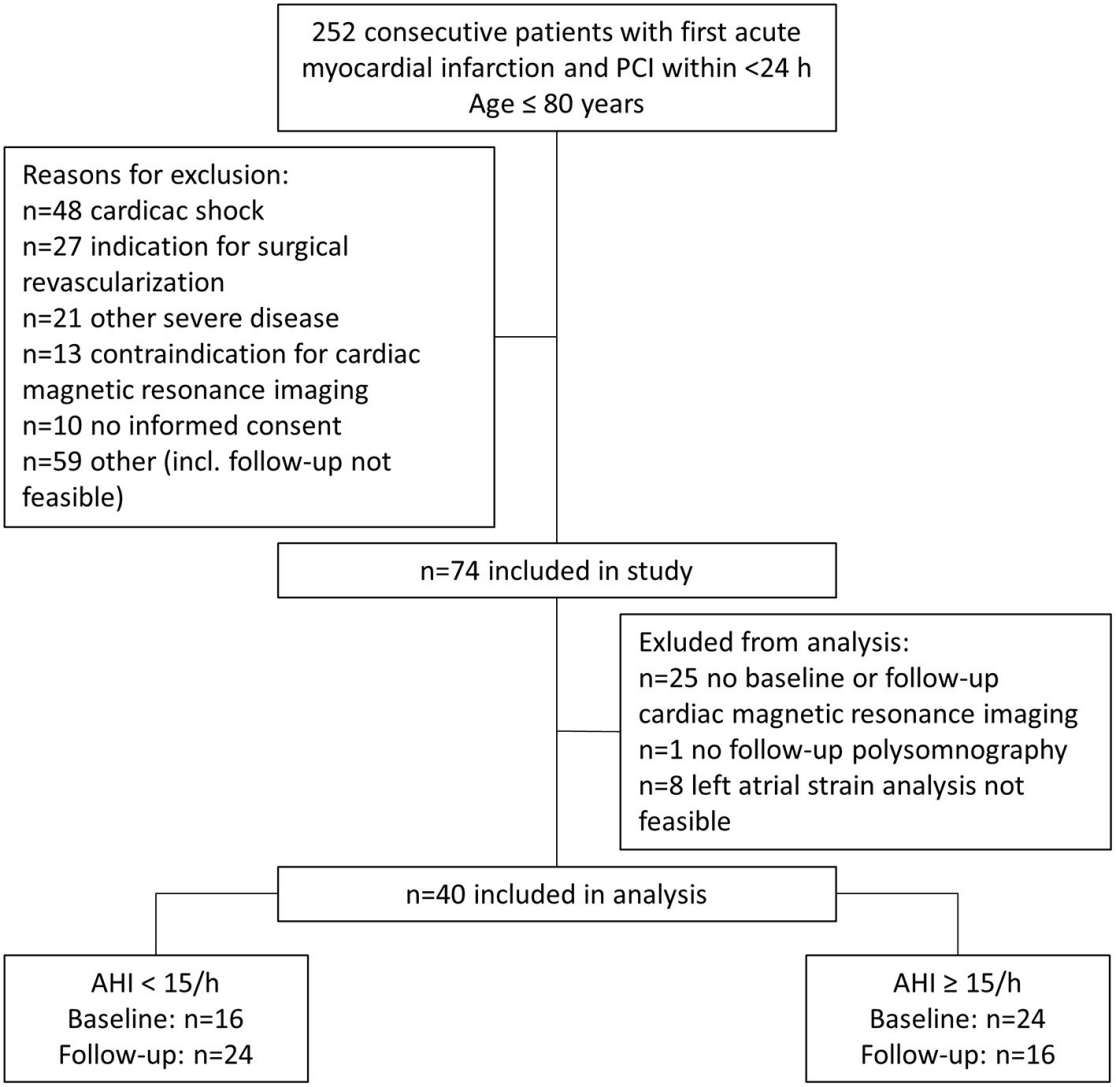
Flow diagram of patients included in the study for analysis at baseline and follow-up. AHI, apnea-hypopnea index; PCI, percutaneous coronary intervention.

After screening, of 252 consecutive patients who underwent percutaneous coronary intervention (March 2009 and March 2012), 74 patients were eligible for the prospective observational study which involved an evaluation of cardiac function and SDB severity at the time of MI (baseline) and 3 months later (follow-up). Thirty four patients were excluded from this sub-analysis due to missing CMR at baseline (*n* = 18) or follow-up (*n* = 7), missing polysomnography at follow-up (*n* = 1), or unfeasible LA strain analysis (*n* = 8). Only patients with complete baseline and follow-up data were included. The final sub-analysis included 40 patients, who were divided into two cohorts depending on AHI at baseline (AHI < 15/h [*n* = 16] and AHI≥15/h [*n* = 24]) and AHI at follow-up (AHI < 15/h [*n* = 24] and AHI≥15/h [*n* = 16]) (Figure 1).

### Polysomnography

All patients underwent polysomnography using standard polysomnographic techniques (Alice System; Respironics, Pittsburgh, PA, USA) as described before (8, 9, 28, 29). Shortly, respiratory efforts were measured by means of respiratory inductance plethysmography, and airflow was measured with a nasal pressure cannula. Sleep stages, arousals, apneas, and hypopneas were analyzed by an experienced sleep technician blinded to the clinical data according to the criteria of the American Academy of Sleep Medicine (30). Apnea was defined as cessation of inspiratory airflow for ≥10 s. Hypopnea definition A was used (≥30% reduction in airflow and ≥4% desaturation) (30). Apnea-hypopnea index (AHI) was defined as the number of central or obstructive apnea and hypopnea episodes per hour of sleep (30). The median time to PSG after acute MI was 3 days.

### CMR Imaging

CMR studies were performed 3–5 days after the MI on a clinical 1.5 Tesla scanner (MAGNETOM; Avanto, Siemens Healthcare Sector, Erlangen, Germany) using a phased array receiver coil during breath-hold and that was ECG-triggered. Cine short-axis and long-axis slices were obtained with a steady-state–free precision gradient-echo sequence (SSFP-sequence; trueFISP; slice thickness 8 mm, inter-slice gap 2 mm, repetition time 60.06 ms, echo time 1.16 ms, flip angle 60°, matrix size 134 x 192 and readout pixel bandwidth 930 Hz pixel^−1^). The number of Fourier lines per heartbeat was adjusted to allow the acquisition of 25 cardiac phases covering systole and diastole within a cardiac cycle. Calculation of cardiac volumes and ejection fraction was performed commercially available software (syngo Argus, version B15; Siemens Healthcare Sector). LV volumes and LV ejection fraction were measured in the serial short axis slices (average of 8 short-axis segments; excluding papillary muscles). Atrial volumes were analyzed in the long axis views.

### CMR Feature Tracking

Atrial myocardial feature tracking was performed semi-automatically using a dedicated software (cvi42, Circle Cardiovascular Imaging Inc., Calgary, Canada). LA borders were manually traced in the 2- and 4-chamber long-axis views using LV end-diastole as the reference phase. If necessary, manual adjustments were performed to obtain optimal wall tracking.

**Figure 2:**
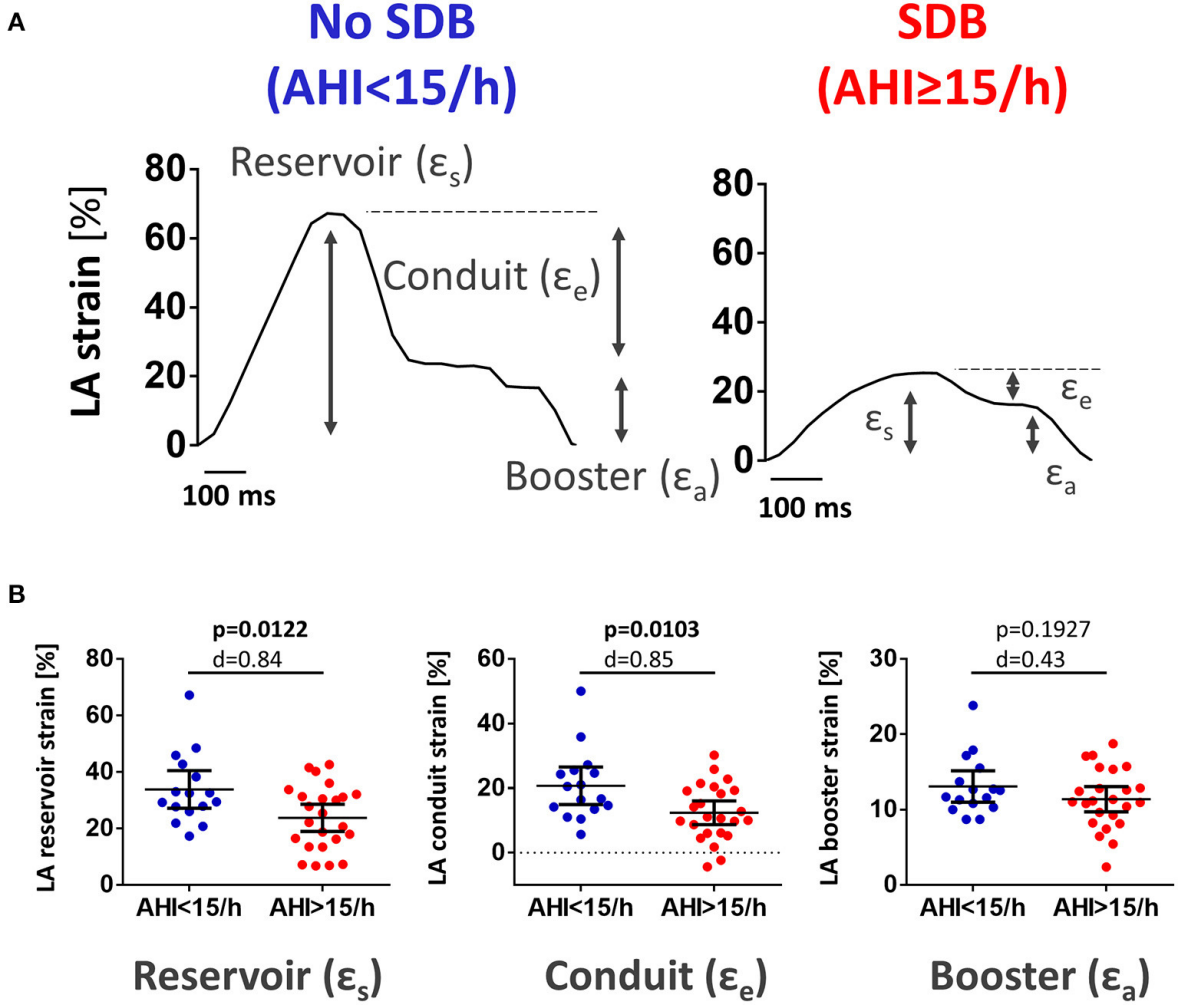
LA reservoir and conduit function were reduced in patients with SDB. **(A)** Original recording of cardiac magnetic resonance imaging feature tracking tracings of left atrial global longitudinal strain in a patient presenting with acute MI without SDB (left) and with SDB (right). Gray arrows indicate the individual components of LA strain: reservoir (ε_s_), conduit (ε_e_), and booster (ε_a_). **(B)** Mean data for LA reservoir, conduit, and booster strain as dichotomized scatter plots. In patients with SDB, there was a significant decrease in LA reservoir and conduit strain. LA booster strain was not different between both groups. Dichotomized scatter plots include *p*-values and Cohen's d.

Analysis of LA myocardial deformation was performed deploying the commonly used Lagrangian strain definition (20). Longitudinal atrial strain was computed as (L_1_-L_0_)/L_0_, where L_0_ is the resting/reference length (LV end diastole) and L_1_ is the change of atrial myocardial length throughout the cardiac cycle. LA strain was analyzed for total strain (ε_s_, corresponding to atrial reservoir function), passive strain (ε_e_, corresponding to atrial conduit function), and active strain (ε_a_, corresponding to atrial booster function) (Figure 2). Strain rate was calculated as change in ε over time: peak positive strain rate (SR_s_, corresponding to atrial reservoir function), peak early negative strain rate (SR_e_, corresponding to atrial conduit function), and peak late negative strain rate (SR_a_, corresponding to atrial booster function) (Supplementary Figure 1).

### Statistical Analysis

Unless indicated otherwise, descriptive data are expressed as means ± SD or as frequencies and percentages of each category. Groups were compared using Student's *T*-test for continuous variables and the Chi-square or Fisher's exact test for categorial variables. Linear regression models were calculated to assess the correlation of AHI and strain as well as with various volumetric and functional LA parameters. Scatter plots with regression lines were used to visualize the relationships between variables. All reported *p*-values are two-sided (except for one-sided *t*-tests for change in AHI from baseline to follow-up in **Figure 4A**). *P*-values < 0.05 were considered statistically significant. Statistical analysis was performed in SPSS (SPSS Statistics for Windows, Version 26.0 Armonk, NY: IBM Corp.) and Graphpad Prism (Version 6.01 for Windows, GraphPad Software, La Jolla California USA).

## Results

### Study Population

Of 252 consecutive patients with MI, 40 were eligible for this analysis (Figure 1). Patient characteristics stratified for an AHI at baseline of <15/h or ≥15/h are shown in Table 1. Patients with SDB (AHI>15/h) had a slightly higher body-mass index (29.65 vs 27.26 kg/m^2^, *p* = 0.006), increased nt-pro BNP levels at discharge (*p* = 0.005), increased left ventricular (LV) mass index (*p* = 0.001). LV ejection fraction was reduced in patients with SDB without reaching statistical significance (*p* = 0.062). Of the 16 patients with no SDB at baseline, two worsened and had SDB at follow-up. Of the 24 patients with SDB at baseline, 10 improved and had no SDB at follow-up. At follow-up, patients with SDB had increased nt-pro-BNP-levels (*p* = 0.028), impaired renal function (*p* = 0.012), decreased LV ejection fraction (*p* = 0.002), and increased values for LV mass index (*p* = 0.036) as well as LV end-diastolic volume (*p* = 0.022, Supplementary Table 1).

**Table 1:** Baseline characteristics stratified for patients with and without SDB (AHI>15/h).

		**AHI <15/h (*N* = 16)**	**AHI≥15/h (*N* = 24)**	
		**Mean ±SD**	**Mean ±SD**	***p*-Value**
Age	[years]	54.18 ± 10.72	56.08 ± 9.75	0.574^T^
Body-mass index	[kg/m^2^]	27.26 ± 1.68	29.65 ± 3.48	**0.006** ^T^
Male	[*n*, %]	13 (81.2%)	22 (91.6%)	0.372^F^
Arterial hypertension	[*n*, %]	9 (56.2%)	14 (58.3%)	0.896^Chi^
Diabetes mellitus	[*n*, %]	2 (12.5%)	5 (20.8%)	0.681^F^
Hypercholesterolemia	[*n*, %]	6 (37.5%)	7 (29.1%)	0.581^Chi^
Current smoking	[*n*, %]	11 (68.8%)	10 (41.7%)	0.117^F^
Apnea-hypopnea-index	[1/h]	6.1 ± 3.36	30.35 ± 14.2	**<0.001** ^T^
STEMI	[*n*, %]	11 (68.8%)	10 (41.7%)	0.117^T^
Creatinine kinase max	[U/L]	1 620.87 ± 1 538.91	2 380.08 ± 1 426.27	0.125^T^
nt-pro-BNP at discharge	[pg/mL]	550.72 ± 410.81	1 377.73 ± 1 227.03	**0.005** ^T^
eGFR	[mL/min/1.73 m^2^]	94.33 ± 16.69	90.37 ± 20.52	0.507^T^
Resting heart rate	[1/min]	72 ± 13.73	79.26 ± 17.19	0.152^T^
Systolic blood pressure	[mmHg]	127.06 ± 25.42	132.45 ± 18.85	0.474^T^
Diastolic blood pressure	[mmHg]	79.62 ± 12.91	79.04 ± 12.1	0.886^T^
LV ejection fraction	[%]	49.42 ± 7.87	44.67 ± 6.89	0.062^T^
LV ejection fraction <35%	[*n*, %]	0 (0%)	2 (8.3%)	0.508^F^
LA area	[cm^2^]	26.31 ± 3.55	24.82 ± 4.24	0.264^T^
LA volume index	[mL/m^2^]	25.60 ± 13.98	28.04 ± 13.50	0.592^T^
LA ejection fraction	[%]	35.43 ± 7.52	29.76 ± 8.74	**0.049** ^T^
LV mass index	[g/m^2^]	68.54 ± 10.98	83.47 ± 14.80	**0.001** ^T^
LV end-diastolic volume	[mL]	152.67 ± 32.52	172.22 ± 40.45	0.104^T^
ACE-inhibitor/angiotensin-receptor blocker	[*n*, %]	15 (93.7%)	24 (100%)	0.400^F^
β-blocker	[*n*, %]	16 (100%)	22 (91.6%)	0.501^F^
Loop diuretics	[*n*, %]	5 (31.2%)	10 (41.6%)	0.740^F^
Mineralocorticoid receptor antagonists	[*n*, %]	7 (43.7%)	14 (58.3%)	0.365^Chi^

*BNP, brain natriuretic peptide; eGFR, estimated glomerular filtration rate; LA, left atrium; LV, left ventricle; SD, standard deviation; SDB, sleep-disordered breathing; STEMI, ST-elevation myocardial infarction. Bold values mean statistical significance calculated by the two-sided Student‘s t-test (T), Chi-Square-test (Chi), or Fisher‘s exact test (F)*.

All patients received similar medication according to current guidelines for the management of acute MI and heart failure (31, 32). Interestingly, only at follow-up systolic and diastolic blood pressure was reduced in patients with SDB (systolic 115 ± 9 mmHg vs 125 ± 14 mmHg; diastolic 68 ± 8 mmHg vs 76 ± 11 mmHg).

### Association Between SDB Severity and Left Atrial Function

**Figure 3:**
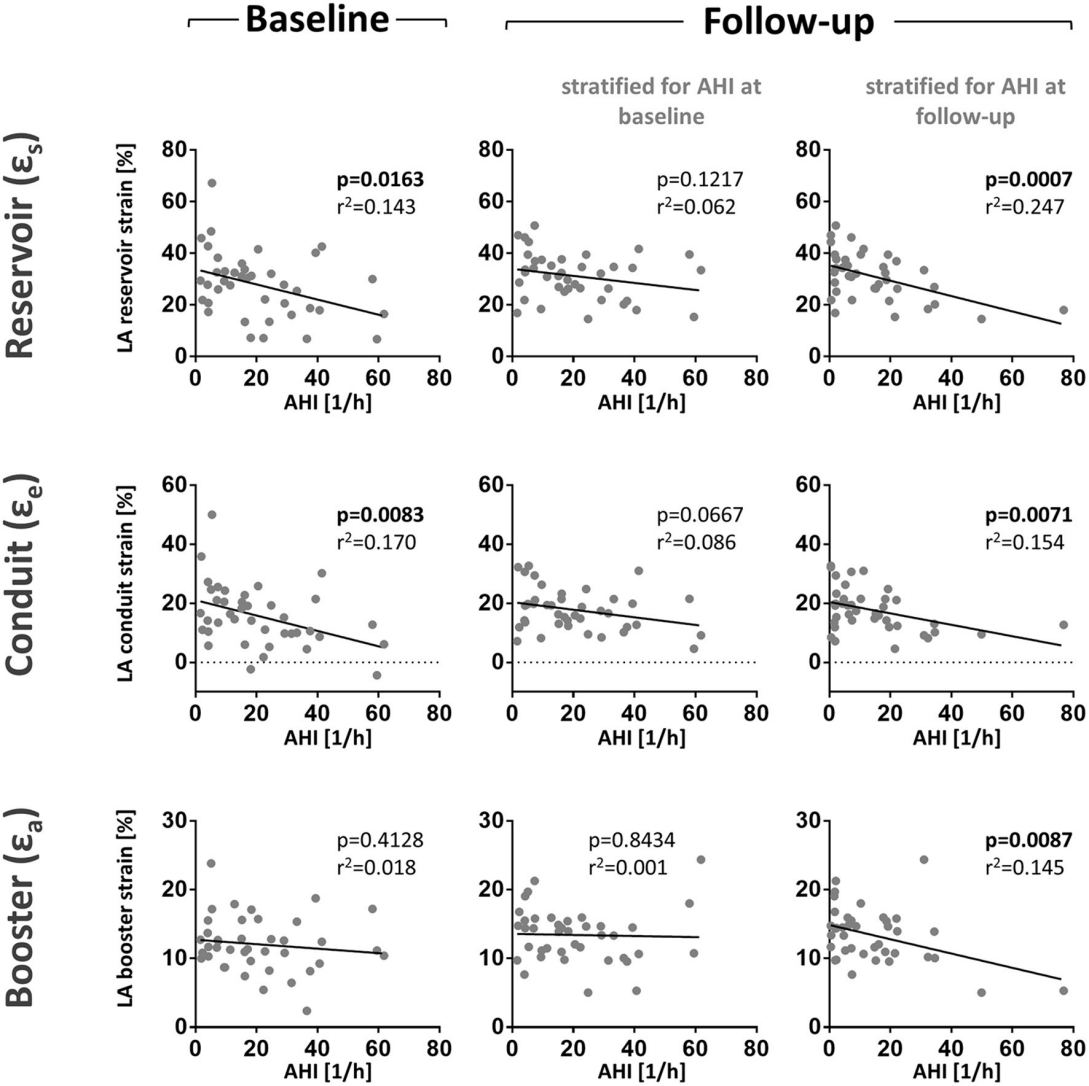
LA function correlates negatively with the apnoe-hypopnoea-index (AHI). Shown are scatter plots of AHI and LA reservoirs, conduit, and booster strain at baseline and follow-up. We performed two analyses for the follow-up data. First, patients were stratified patients for AHI measured at baseline to evaluate the intra-individual development of SDB and strain over time (middle panel). In this stratification, the correlation of AHI and LA reservoir strain was lost at follow-up. Second, patients were stratified for AHI measured during follow-up to assess the influence of AHI on LA strain (right panel). In this stratification, the negative correlation between AHI and La reservoir strain was highly significant. These results show that the AHI may be the main predictor of LA reservoir strain in contrast to other individual factors. Correlation scatter plots include *p*-values (bold letters signify statistical significance *p* < 0.05) and adjusted R^2^-values.

Interestingly, during the hospitalization for acute MI (i.e., baseline), patients with SDB exhibited significantly lower LA strain and strain rate as a measure of LA reservoir and conduit function (Figure 2; Supplementary Figure 1). There was also a significant negative correlation between AHI as measure of SDB severity and the magnitude of LA reservoir and conduit strain and strain rate suggesting a causal role of SDB in impaired LA function (Figure 3; Supplementary Figure 2). In contrast, LA booster function was not affected by SDB (Figure 3; Supplementary Figure 2).

We then tested if strain analysis could provide a more detailed evaluation of atrial function than conventional volumetric LA parameters. Intriguingly, there was no correlation of LA area and AHI (Table 2) possibly because of hemodynamic confounding due to LV dysfunction. In fact, LV dysfunction correlated significantly with AHI (Table 2).

**Table 2:** Linear regression of AHI with volumetric LA parameters and volumetric ventricular parameters.

	**Linear regression analysis at baseline**				**Linear regression analysis at follow-up**							
	**With AHI at baseline**				**With AHI at baseline**				**With AHI at follow-up**			
	**B**	**95% CI**	**R^**2**^ (adj.)**	***P* value**	**B**	**95% CI**	**R^**2**^ (adj.)**	***P* value**	**B**	**95% CI**	**R^**2**^ (adj.)**	***P* value**
LA diastolic area	0.625	−2.330 to 1.080	−0.013	0.461	−0.134	−6.224 to 55.100	−0.028	0.863	1.290	−0.120 to 2.690	0.065	0.072
LA volume index	0.180	−0.185 to 0.623	0.006	0.279	−0.120	−0.640 to 0.303	−0.013	0.473	−0.060	−0.538 to 0.375	−0.024	0.718
LV ejection fraction	−0.800	−1.500 to−0.110	0.107	**0.025**	−0.717	27.633 to 86.196	0.127	**0.015**	−0.840	−1.340 to−0.330	0.217	**0.002**

*AHI, apnea-hypopnea-index; CI, confidence interval; LA, left atrium; LV, left ventricle. Bold values mean statistical significance p < 0.05*.

### Association Between SDB and Left Atrial Strain at Follow-Up

We then sought to evaluate the time course of the association between SDB severity and left atrial function from baseline to the 3-month follow-up as this might provide insights into the causal relationship between SDB and LA function. Therefore, we performed two analyses for the follow-up data. First, we stratified patients for AHI measured at baseline to evaluate the intra-individual development of SDB and strain over time. Interestingly, the association between SDB and reduced atrial function being it LA reservoir or conduit (Figure 3) was substantially weaker compared to the baseline analysis (Figure 3). However, if patients were stratified for AHI measured during follow-up, the association of follow-up LA strain with follow-up AHI was of comparable magnitude as to association at baseline. Importantly, in contrast to LA booster at baseline, the magnitude of LA booster at follow-up correlated significantly negative with follow-up AHI (Figure 3).

**Figure 4:**
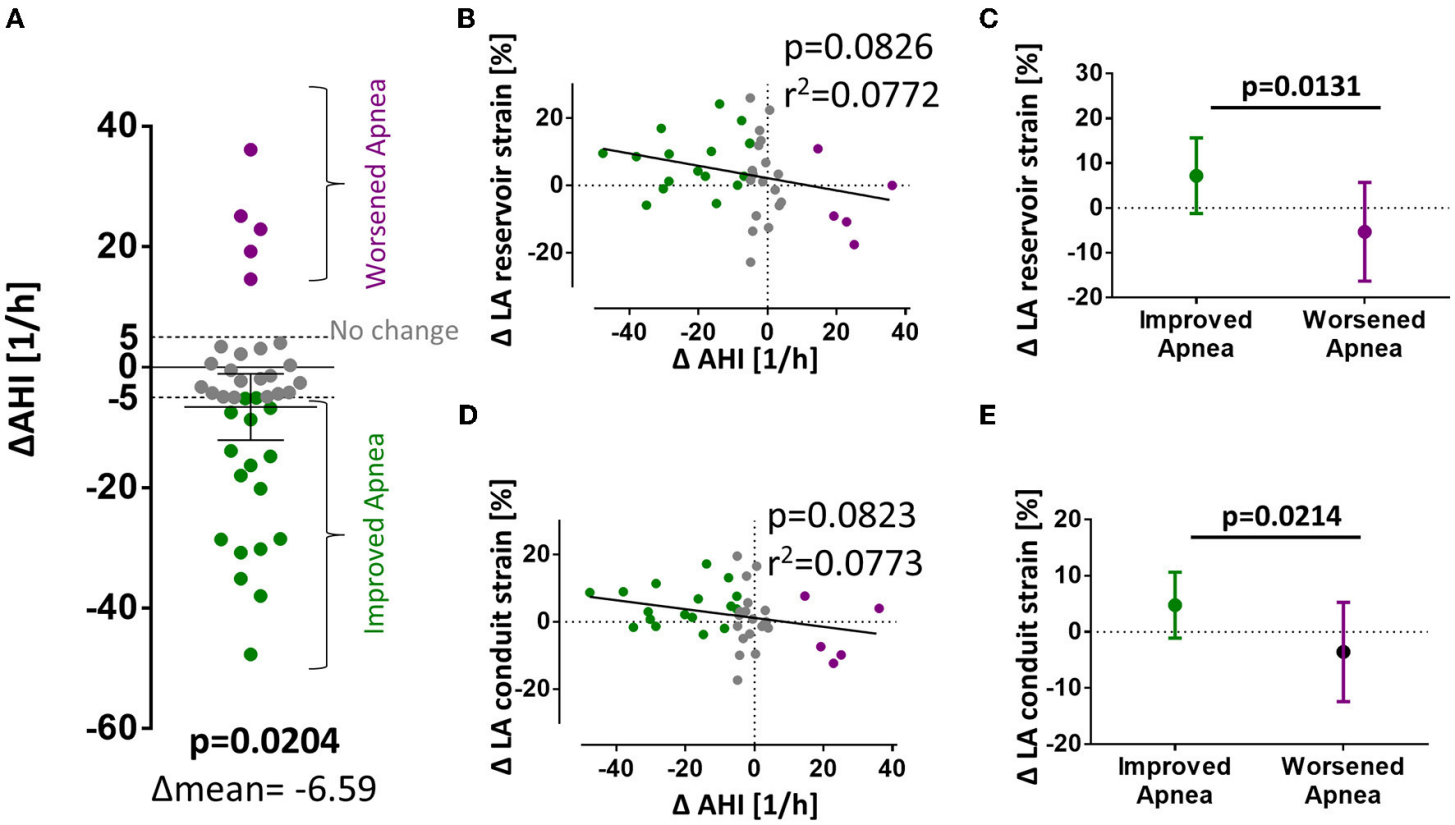
Changes in AHI and LA strain from baseline to follow-up. **(A)** Significant reduction of AHI between baseline and follow-up. **(B–E)** Show differences in strain values from baseline to follow-up organized vertically for LA ε_s_, ε_e_, and ε_a_ and horizontally for mean difference; correlation of differences in AHI and strain; and strain values dichotomized for reduction or increase in AHI (> ±5/h, respectively). *p*-values for total mean difference are calculated with the one-sided Student's *t*-test. Comparison between dichotomized groups were calculated with the two-sided Student's *t*-test. Bold *p*-values signify statistical significance *p* < 0.05.

A possible explanation for the different association of LA strain with AHI at baseline and follow-up may result from a different frequency of apnea events during both time points. In fact, there was a significant reduction in mean AHI from baseline to follow-up (Figure 4A).

To test if a change in AHI may be causally linked to a change in LA strain, we performed a correlation of the ΔAHI with the ΔLA reservoir and ΔLA conduit function which showed a strong negative trend but missed statistical significance (linear regression, *p* = 0.08 and B = −0.1834 for ε_s_; *p* = 0.08 and B = −0.1316 for ε_e_, Figures 4B,D). As a rule of thumb, in our model, an improvement of AHI of 10/h is linked to an improvement in LA reservoir function of +1.8 percentage points and of +1.3 percentage points in LA conduit function. After dichotomization, patients with improved SDB from baseline to follow-up (ΔAHI < −5/h) were significantly more likely to develop improved atrial strain (Figure 4C). Patients with improved SDB (ΔAHI < −5/h) exhibited a mean improvement of LA reservoir strain of +7.2 ± 8.4% whereas patients with SDB deterioration (ΔAHI> +5/h) showed a mean decrease of −5.3 ± 11.0% (*p* = 0.0131) (Figures 4C,E).

## Discussion

Our data shows that the measurement of atrial strain by feature tracking analysis of CMR imaging provides a more sensitive and robust marker for atrial function than conventional volumetric LA parameters. At the time of MI and at the 3-month follow-up, atrial function was decreased in patients with SDB. Moreover, the change in SDB severity from baseline to follow-up correlated with the change in atrial strain function, suggesting a close causal connection between SDB severity and atrial function.

### Atrial Strain Is a Robust Marker for Atrial Dysfunction After Ischemic Damage in Patients With SDB

Ischemic damage in MI mainly involves both ventricles depending on the culprit lesion. Atrial damage in MI may mostly be secondary to the impaired ventricular function and systemic effects during MI such as increased activation of the sympathetic nervous system and of inflammatory pathways. However, direct hypoxic damage in the atria may be present more frequently than is generally suspected, as histopathological studies have reported the incidence of concomitant atrial infarction in 0.7% to 42% of cases (33). Atrial and ventricular hemodynamics are closely connected and mutually dependent (34). On the one hand, passive LA function suffers from LV impairment, and on the other hand, LA size, contractility, and compliance are important for optimal LV filling (34). Therefore, it seems plausible that contractile impairment of the left ventricle upon MI would also result in atrial contractile dysfunction as well as that any atrial damage might further reduce LV function and cardiac output. This atrial damage may be aggravated by concomitant SDB both acutely and chronically.

Currently, assessment of the LA function is routinely performed by volumetric measurement of LA size or volume (34). However, those parameters do not directly describe atrial function. In our cohort, diastolic LA area and LA volume index did neither correlate with AHI at baseline nor at follow-up (stratified for AHI at baseline or stratified for AHI at follow-up) (Table 2). Current guidelines for echocardiographic LA quantification do not include measurement of LA ejection fraction as a functional parameter due to methodological difficulties (17). In contrast to these conventional parameters of atrial function, LA strain analysis in CMR cine data offers a reliable tool to measure atrial function in healthy individuals (22). In patients with acute myocarditis, reduced LA strain has been found to be a sensitive parameter of atrial dysfunction (23). To our knowledge, the effect of SDB on atrial function assessed by CMR-derived atrial strain has not yet been implored. Here, we show that patients with severe SDB (AHI>15/h) exhibit significantly impaired atrial strain upon acute MI. Moreover, AHI also negatively correlates with LA function (Figure 3; Supplementary Figure 2) suggesting a cause-effect relationship between the severity of sleep apnea and LA dysfunction.

Our group has shown that SDB is associated with reduced LV ejection fraction and that SDB negatively affects cardiac remodeling in this cohort. There was a clinically relevant reduction in the severity of SDB in patients whose LV ejection fraction improved in the 12 weeks following myocardial infarction whereas the AHI was unchanged in patients whose LV ejection did not improve (27). The salvage index was reduced in patients with SDB and consequentially, the infarct size was significantly larger at follow-up in patients with SDB (8). Also, structural remodeling was negatively affected by obstructive sleep apnea (9). It might be suspected that SDB would also negatively affect atrial function over the time course from baseline to follow-up. We have therefore also analyzed the longitudinal change in LA strain after 3 months of follow-up. Intriguingly, we could show that the association between the initial AHI measured during MI and atrial strain measured after 3 months was lost (Figure 3; Supplementary Figure 2). However, the AHI measured during follow-up significantly correlated with LA function at follow-up (Figure 3; Supplementary Figure 2). These observations suggest that that LA function was not yet permanently impaired, but rather that SDB has a direct influence on atrial function and that short-term fluctuations in SDB severity or AHI may produce corresponding changes in atrial function.

In accordance, the magnitude of the time-dependent change in AHI from initial investigation to follow-up correlated with the magnitude of atrial function (Figures 3B,D). Unfortunately, there are variations in AHI values between consecutive polysomnographies that are inherent to the method. Chediak and colleagues reported a mean AHI difference of 3/h in two consecutive nights (35) and Levendowski and colleagues found a mean difference for the AHI of 6/h when the two measurements were 1 month apart (36). Based on the above mentioned empirical data, we determined changes in AHI > ±5/h between baseline and 3-month follow-up to be clinically significant and stratified patients for improvement, deterioration, or no difference of SDB severity. According to this stratification, a statistically significant difference between atrial strain values (reservoir and conduit) in patients with worsened or improved AHI can be observed (Figures 3C,E).

There are some circumstantial factors that have to be considered when comparing the measurements for AHI and atrial strain at baseline and follow-up. First, MI leads to acute heart failure and abrupt changes in LV hemodynamics which instantly affect atrial function. Reduced LV ejection fraction results in reduced displacement of the atrioventricular plane which is a major contributor to passive LA reservoir function. Thus, reduced LV ejection fraction contributes to reduced LA reservoir function. Reduced LV ejection fraction and forward heart failure elevate atrial pressure which also affects atrial function. This notion is supported by our observation of increased nt-pro-BNP levels for patients with SDB and reduced atrial strain at baseline (Table 1). Second, SDB is associated with reduced LV ejection fraction and the burden of SDB is reduced when the LV function recovers after myocardial infarction (27). These observations suggest that the AHI measured at the initial hospital admission during MI might be overestimated and that patients that would normally not qualify for SDB exhibit an increased AHI which then, over the time to the follow-up, normalizes again. This view is strengthened by the shifting percentage of patients with an AHI >15/h that drops from initially 60% (24 of 40) to 40% (16 of 40) at follow-up (Figure 1).

Despite these considerations, our data shows a moderate correlation of AHI and LA function at two different points in time, thus strongly suggesting that SDB is connected to LA dysfunction in the acute and chronic setting of cardiac disease.

### Study Limitations

All CMR cine data were captured in three different planes in the two- and four chamber views. For the calculation of strain, the plane best depicting atrial geometry was chosen. However, the strain analysis was performed on cine images that were originally intended to provide optimal quantification of the left ventricle. Thus, the geometry of the atria might not be optimally represented, and the strain be miscalculated. This was the case in eight patients which have therefore been excluded from the analysis. A selection bias toward less severe cases may occur due to the exclusion criteria which excluded severe lung disease and treated SDB.

## Conclusion

Our results show that LA function measured by CMR strain analysis is impaired in patients with SDB both chronically and during states of acute cardiac injury such as MI. Correlation between AHI and strain values was more robust at follow-up suggesting that disruptive factors may obscure the relationship of SDB and atrial dysfunction in states of acute cardiac injury. Interestingly, conventional volumetric parameters of atrial function such as LA area and LA volume index did not correlate with AHI at baseline or follow-up. LA strain measurement thus provides additional insight into atrial function at acute and chronic cardiac injury and SDB.

## Clinical Outlook

“Sleep-disordered breathing and especially obstructive sleep apnea are highly prevalent and clinically relevant cardiovascular risk factors. As they result in impaired cardiac and particularly atrial function, it is crucial to identify patients at risk with polysomnography. Our group has recently shown that the magnitude of the ECG parameter P-wave terminal force in lead V1 is associated with the apnea-hypopnea index and may thus serve as a readily available parameter to identify patients that should be screened for SDB by polysomnography (37). Treatment options for obstructive sleep apnea include automatic positive airway pressure, which has recently been shown to improve cardiopulmonary exercise capacity and left ventricular ejection fraction in patients with heart failure with reduced ejection fraction (38). The results of this observational study reported here have led to the initiation of the interventional TEAM-ASV-I trial (39). It is, therefore, crucial to efficiently screen cardiac patients to identify and treat sleep-disordered breathing.

## Data Availability Statement

The raw data supporting the conclusions of this article will be made available by the authors, without undue reservation.

## Ethics Statement

This study involving human participants was reviewed and approved by Ethikkommission an der Universität Regensburg. The patients/participants provided their written informed consent to participate in this study.

## Author Contributions

MW and SW designed this post-hoc analysis. KD, OH, FP, SB, and MA enrolled participants and acquired the data. MW, JP, SL, CF, KD, OH, FP, and SB analyzed the study data. MW wrote the initial draft of the manuscript. All authors were involved at all stages of the critical revision of the manuscript, read and approved the final manuscript, and meet the criteria for authorship as recommended by the International Committee of Medical Journal Editors.

## Funding

MW, SL, and CF are supported by the local ReForM-program. SW is funded by DFG grants WA 2539/7-1 and 8-1. LM is funded by DFG grants MA 1982/7-1. SW and LM are also supported by the DFG SFB 1350 grant (Project Number 387509280, TPA6). MA received grant support from the Else-Kroener Fresenius Foundation (2018_A159). CF received a grant from the German Heart Foundation/German Foundation of Heart Research (F/15/20; CF).

## Conflict of Interest

MA received lecture and consulting fees from ResMed, Philips Respironics, Boehringer-Ingelheim, NRI, Novartis, JAZZ pharmaceuticals, Bayer, Inspire and Bresotec and grant support from ResMed Foundation, ResMed, and Philips Respironics outside the submitted work. The remaining authors declare that the research was conducted in the absence of any commercial or financial relationships that could be construed as a potential conflict of interest.

## Publisher's Note

All claims expressed in this article are solely those of the authors and do not necessarily represent those of their affiliated organizations, or those of the publisher, the editors and the reviewers. Any product that may be evaluated in this article, or claim that may be made by its manufacturer, is not guaranteed or endorsed by the publisher.

## Abbreviations

AHI: apnea-hypopnea-index
CMR: cardiac magnetic resonance imaging
CI: confidence interval
eGFR: estimated glomerular filtration rate
LA: left atrium
LV: left ventricle
MI: myocardial infarction
SDB: sleep-disordered breathing
SD: standard deviation
STEMI: ST-elevation myocardial infarction
ε: strain
SR: strain rate.

## References

[1] Benjafield AV Ayas NT Eastwood PR Heinzer R Ip MSM Morrell MJ. Estimation of the global prevalence and burden of obstructive sleep apnoea: a literature-based analysis. Lancet Respir Med. (2019) 7:687–98. 10.1016/S2213-2600(19)30198-5 31300334 PMC7007763

[2] Floras JS. Sleep Apnea and Cardiovascular Disease: An Enigmatic Risk Factor. Circ Res. (2018) 122:1741–64. 10.1161/CIRCRESAHA.118.310783 29880501

[3] Tkacova R Rankin F Fitzgerald FS Floras JS Bradley TD. Effects of continuous positive airway pressure on obstructive sleep apnea and left ventricular afterload in patients with heart failure. Circulation. (1998) 98:2269–75. 10.1161/01.CIR.98.21.2269 9826313

[4] Nakashima H Katayama T Takagi C Amenomori K Ishizaki M Honda Y. Obstructive sleep apnoea inhibits the recovery of left ventricular function in patients with acute myocardial infarction. Eur Heart J. (2006) 27:2317–22. 10.1093/eurheartj/ehl219 16956914

[5] Lee CH Khoo SM Tai BC Chong EY Lau C Than Y. Obstructive sleep apnea in patients admitted for acute myocardial infarction Prevalence, predictors, and effect on microvascular perfusion. Chest. (2009) 135:1488–95. 10.1378/chest.08-2336 19497895

[6] Oldenburg O Lamp B Faber L Teschler H Horstkotte D Töpfer V. Sleep-disordered breathing in patients with symptomatic heart failure: a contemporary study of prevalence in and characteristics of 700 patients. Eur J Heart Fail. (2007) 9:251–7. 10.1016/j.ejheart.2006.08.003 17027333

[7] Schulz R Blau A Börgel J Duchna HW Fietze I Koper I. Sleep apnoea in heart failure. Eur Respir J. (2007) 29:1201–5. 10.1183/09031936.00037106 17360729

[8] Buchner S Satzl A Debl K Hetzenecker A Luchner A Husser O. Impact of sleep-disordered breathing on myocardial salvage and infarct size in patients with acute myocardial infarction. Eur Heart J. (2014) 35:192–9. 10.1093/eurheartj/eht450 24164862

[9] Fisser C Götz K Hetzenecker A Debl K Zeman F Hamer OW. Obstructive sleep apnoea but not central sleep apnoea is associated with left ventricular remodelling after acute myocardial infarction. Clin Res Cardiol. (2021) 110:971–82. 10.1007/s00392-020-01684-z 32519084 PMC8238704

[10] Gerdts E Wachtell K Omvik P Otterstad JE Oikarinen L Boman K. Left atrial size and risk of major cardiovascular events during antihypertensive treatment: losartan intervention for endpoint reduction in hypertension trial. Hypertension. (2007) 49:311–6. 10.1161/01.HYP.0000254322.96189.85 17178978

[11] Hoit BD. Left atrial size and function: role in prognosis. J Am Coll Cardiol. (2014) 63:493–505. 10.1016/j.jacc.2013.10.055 24291276

[12] Overvad TF Nielsen PB Larsen TB Søgaard P. Left atrial size and risk of stroke in patients in sinus rhythm. A systematic review. Thromb Haemost. (2016) 116:206–19. 10.1160/TH15-12-0923 27075168

[13] Roeder M von Rommel KP Kowallick JT Blazek S Besler C Fengler K. Influence of Left Atrial Function on Exercise Capacity and Left Ventricular Function in Patients With Heart Failure and Preserved Ejection Fraction. Circ Cardiovasc Imaging. (2017) 10:e012468. 10.1161/CIRCIMAGING.117.006785 28360259

[14] Moller JE Hillis GS Oh JK Seward JB Reeder GS Wright RS. Left atrial volume: a powerful predictor of survival after acute myocardial infarction. Circulation. (2003) 107:2207–12. 10.1161/01.CIR.0000066318.21784.43 12695291

[15] Hohl M Linz B Böhm M Linz D. Obstructive sleep apnea and atrial arrhythmogenesis. Curr Cardiol Rev. (2014) 10:362–8. 10.2174/1573403X1004140707125137 25004989 PMC4101201

[16] Sascău R Zota IM Stătescu C Boişteanu D Roca M Maştaleru A. Review of echocardiographic findings in patients with obstructive sleep Apnea. Can Respir J. (2018) 2018:1206217. 10.1155/2018/1206217 30581512 PMC6276396

[17] Lang RM Badano LP Mor-Avi V Afilalo J Armstrong A Ernande L. Recommendations for cardiac chamber quantification by echocardiography in adults: an update from the American Society of Echocardiography and the European Association of Cardiovascular Imaging. Eur Heart J Cardiovasc Imaging. (2015) 16:233–70. 10.1093/ehjci/jev014 25712077

[18] Kim SM Cho KI Kwon JH Lee HG Kim TI. Impact of obstructive sleep apnea on left atrial functional and structural remodeling beyond obesity. J Cardiol. (2012) 60:475–83. 10.1016/j.jjcc.2012.07.007 22890070

[19] Altekin RE Yanikoglu A Karakas MS Ozel D Kucuk M Yilmaz H. Assessment of left atrial dysfunction in obstructive sleep apnea patients with the two dimensional speckle-tracking echocardiography. Clin Res Cardiol. (2012) 101:403–13. 10.1007/s00392-011-0404-2 22222546

[20] Scatteia A Baritussio A Bucciarelli-Ducci C. Strain imaging using cardiac magnetic resonance. Heart Fail Rev. (2017) 22:465–76. 10.1007/s10741-017-9621-8 28620745 PMC5487809

[21] Pathan F Zainal Abidin HA Vo QH Zhou H D'Angelo T Elen E. Left atrial strain: a multi-modality, multi-vendor comparison study. Eur Heart J Cardiovasc Imaging. (2021) 22:102–10. 10.1093/ehjci/jez303 31848575

[22] Kowallick JT Kutty S Edelmann F Chiribiri A Villa A Steinmetz M. Quantification of left atrial strain and strain rate using Cardiovascular Magnetic Resonance myocardial feature tracking: a feasibility study. J Cardiovasc Magn Reson. (2014) 16:60. 10.1186/s12968-014-0060-6 25196447 PMC4422260

[23] Dick A Schmidt B Michels G Bunck AC Maintz D Baeßler B. Left and right atrial feature tracking in acute myocarditis: a feasibility study. Eur J Radiol. (2017) 89:72–80. 10.1016/j.ejrad.2017.01.028 28267553

[24] Backhaus SJ Stiermaier T Lange T Chiribiri A Uhlig J Freund A. Atrial mechanics and their prognostic impact in Takotsubo syndrome: a cardiovascular magnetic resonance imaging study. Eur Heart J Cardiovasc Imaging. (2019) 20:1059–69. 10.1093/ehjci/jey219 30649241

[25] Chirinos JA Sardana M Ansari B Satija V Kuriakose D Edelstein I. Left atrial phasic function by cardiac magnetic resonance feature tracking is a strong predictor of incident cardiovascular events. Circ Cardiovasc Imaging. (2018) 11:e007512. 10.1161/CIRCIMAGING.117.007512 30562112 PMC6301081

[26] Huber AT Lamy J Rahhal A Evin M Atassi F Defrance C. Cardiac MR strain: a noninvasive biomarker of fibrofatty remodeling of the left atrial myocardium. Radiology. (2018) 286:83–92. 10.1148/radiol.2017162787 28813234

[27] Buchner S Greimel T Hetzenecker A Luchner A Hamer OW Debl K. Natural course of sleep-disordered breathing after acute myocardial infarction. Eur Respir J. (2012) 40:1173–9. 10.1183/09031936.00172211 22441744

[28] Buchner S Eglseer M Debl K Hetzenecker A Luchner A Husser O. Sleep disordered breathing and enlargement of the right heart after myocardial infarction. Eur Respir J. (2015) 45:680–90. 10.1183/09031936.00057014 25359347

[29] Fisser C Marcinek A Hetzenecker A Debl K Luchner A Sterz U. Association of sleep-disordered breathing and disturbed cardiac repolarization in patients with ST-segment elevation myocardial infarction. Sleep Med. (2017) 33:61–7. 10.1016/j.sleep.2017.01.007 28449908

[30] Berry RB Budhiraja R Gottlieb DJ Gozal D Iber C Kapur VK. Rules for scoring respiratory events in sleep: update of the 2007 AASM Manual for the Scoring of Sleep and Associated Events. Deliberations of the Sleep Apnea Definitions Task Force of the American Academy of Sleep Medicine. J Clin Sleep Med. (2012) 8:597–619. 10.5664/jcsm.2172 23066376 PMC3459210

[31] Ponikowski P Voors AA Anker SD Bueno H Cleland JGF Coats AJS. 2016 ESC Guidelines for the diagnosis and treatment of acute and chronic heart failure: The Task Force for the diagnosis and treatment of acute and chronic heart failure of the European Society of Cardiology (ESC)Developed with the special contribution of the Heart Failure Association (HFA) of the ESC. Eur Heart J. (2016) 37:2129–200. 10.1093/eurheartj/ehw128 27206819

[32] Ibanez B James S Agewall S Antunes MJ Bucciarelli-Ducci C Bueno H. 2017 ESC Guidelines for the management of acute myocardial infarction in patients presenting with ST-segment elevation: the task force for the management of acute myocardial infarction in patients presenting with ST-segment elevation of the European Society of Cardiology (ESC). Eur Heart J. (2018) 39:119–77. 10.1093/eurheartj/ehx393 28886621

[33] Lu MLR Venecia T de Patnaik S Figueredo VM. Atrial myocardial infarction: a tale of the forgotten chamber. Int J Cardiol. (2016) 202:904–9. 10.1016/j.ijcard.2015.10.070 26485186

[34] Blume GG Mcleod CJ Barnes ME Seward JB Pellikka PA Bastiansen PM. Left atrial function: physiology, assessment, and clinical implications. Eur J Echocardiogr. (2011) 12:421–30. 10.1093/ejechocard/jeq175 21565866

[35] Chediak AD Acevedo-Crespo JC Seiden DJ Kim HH Kiel MH. Nightly variability in the indices of sleep-disordered breathing in men being evaluated for impotence with consecutive night polysomnograms. Sleep. (1996) 19:589–92. 10.1093/sleep/19.7.589 8899939

[36] Levendowski DJ Zack N Rao S Wong K Gendreau M Kranzler J. Assessment of the test-retest reliability of laboratory polysomnography. Sleep Breath. (2009) 13:163–7. 10.1007/s11325-008-0214-6 18766393

[37] Pec J Wester M Fisser C Debl K Hamer OW Poschenrieder F. Central Sleep Apnea Is Associated with an Abnormal P-Wave Terminal Force in Lead V1 in Patients with Acute Myocardial Infarction Independent from Ventricular Function. J Clin Med. (2021) 10:5555. 10.3390/jcm10235555 34884253 PMC8658572

[38] Fox H Bitter T Sauzet O Rudolph V Oldenburg O. Automatic positive airway pressure for obstructive sleep apnea in heart failure with reduced ejection fraction. Clin Res Cardiol 2021; 110:983–992. 10.1007/s00392-020-01701-1 32651657 PMC8238771

[39] Fox H Hetzenecker A Stadler S Oldenburg O Hamer OW Zeman F. Rationale and design of the randomised Treatment of sleep apnoea Early After Myocardial infarction with Adaptive Servo-Ventilation trial (TEAM-ASV I). Trials. (2020) 21:129. 10.1186/s13063-020-4091-z 32005277 PMC6995094

