# Supplementary material for: Sleep-Disordered Breathing Is Associated With Reduced Left Atrial Strain Measured by Cardiac Magnetic Resonance Imaging in Patients After Acute Myocardial Infarction
Source: Front Med (Lausanne). 2022 Feb 16;9:759361. doi: 10.3389/fmed.2022.759361 (PMC8888827; doi:10.3389/fmed.2022.759361)
Supplement: Supplementary file 1 [file Data_Sheet_1.docx]

# **Supplement Figures**

**Supplement figure 1: LA reservoir and conduit strain rate were reduced in patients with SDB.** A) Original recording of cardiac magnetic resonance imaging feature tracking tracings of left atrial global longitudinal strain rate in a patient presenting with acute MI without SDB (left) and with SDB (right). Grey arrows indicate the individual components of LA strain rate: reservoir (SRs), conduit (SRe), and booster (SRa). B) Mean data for LA reservoir, conduit, and booster strain rate as dichotomized scatter plots. In patients with SDB, there was a significant decrease in LA reservoir and conduit strain rate. LA booster strain rate was not different between both groups. Dichotomized scatter plots include p-values and Cohen’s d. Correlation scatter plots include p-values (bold letters signify statistical significance p<0.05) and adjusted r^2^-values.


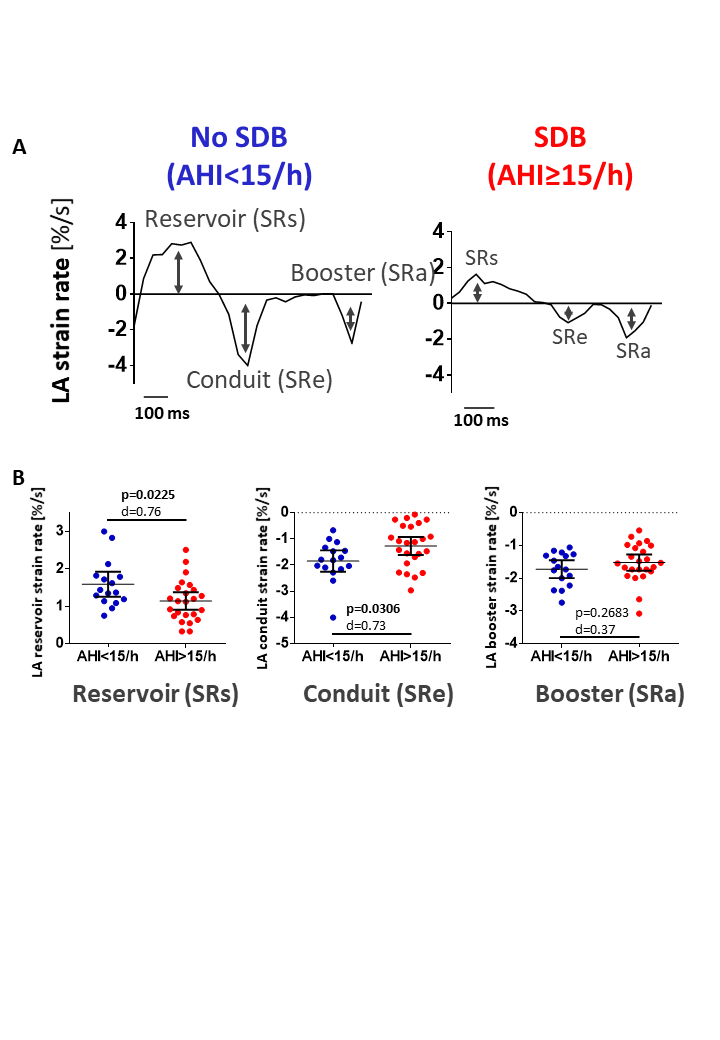


**Supplement figure 2: LA strain rate correlates negatively with the apnoe-hypopnoea-index (AHI).** Shown are scatter plots of AHI and LA reservoirs, conduit, and booster strain rate at baseline and follow-up. We performed two analyses for the follow-up data. First, patients were stratified patients for AHI measured at baseline to evaluate the intra-individual development of SDB and strain over time (middle panel). In this stratification, the correlation of AHI and LA reservoir and conduit strain rate was lost at follow-up. Second, patients were stratified for AHI measured during follow-up to assess the influence of AHI on LA strain rate (right panel). In this stratification, the correlation between AHI and LA reservoir and conduit strain rate was highly significant. These results show that the AHI may be the main predictor of LA reservoir strain rate in contrast to other individual factors. Correlation scatter plots include p-values (bold letters signify statistical significance p<0.05) and adjusted r^2^-values.


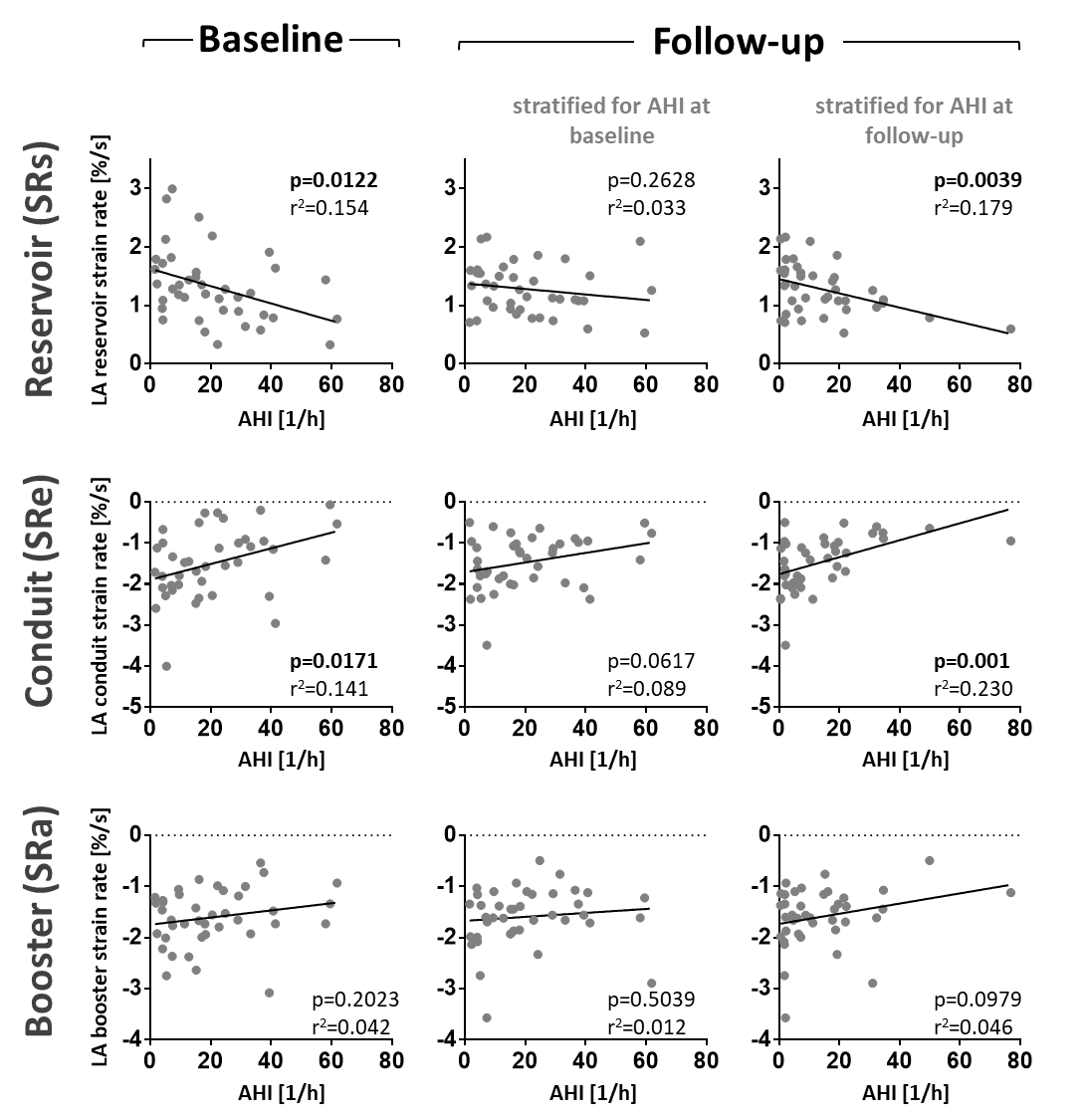


# **Supplement Tables**

**Supplemental table 1:** **Patient characteristics stratified for patients with and without SDB at follow-up (AHI>15/h).**

|  |  | **AHI<15/h**  (N=24) | **AHI>15/h**  (N=16) |  |
| --- | --- | --- | --- | --- |
|  |  | Mean ± SD | Mean ± SD | p-Value |
| Age | [years] | 52.95±9.27 | 58.87±10.44 | 0.067^T^ |
| Body-mass index | [kg/m^2^] | 27.89±2.66 | 29.7±3.6 | 0.079^T^ |
| Male | [n, %] | 21(87.5%) | 14(87.5%) | 1.00^F^ |
| Arterial hypertension^¶^ | [n, %] | 15(62.5%) | 8(50%) | 0.433^Chi^ |
| Diabetes mellitus^¶^ | [n, %] | 5(20.8%) | 2(12.5%) | 0.680^F^ |
| Hypercholesterolemia^¶^ | [n, %] | 11(45.8%) | 2(12.5%) | **0.040**^F^ |
| Current smoking^¶^ | [n, %] | 12(50%) | 9(56.2%) | 0.698^Chi^ |
| Apnea-hypopnea index | [1/h] | 4.7±3.82 | 28.1±15.96 | **<0.001**^T^ |
| STEMI^¶^ | [n, %] | 13(54.2%) | 8(50.0%) | 1.000^F^ |
| Creatinine kinase max at MI^¶^ | [U/L] | 1727.5±1546.58 | 2599.75±1304.52 | 0.071^T^ |
| nt-pro-BNP | [pg/mL] | 285.31±269.45 | 885.55±1194.34 | **0.028**^T^ |
| eGFR | [mL/min/  1,73 m^2^] | 94.75±15.26 | 79.29±21.35 | **0.012**^T^ |
| Resting heart rate | [1/min] | 64.79±9.81 | 64.26±7.16 | 0.858^T^ |
| Systolic blood pressure | [mmHg] | 124.62±13.61 | 115.42±8.93 | **0.030**^T^ |
| Diastolic blood pressure | [mmHg] | 76.04±10.75 | 68.42±8.24 | **0.028**^T^ |
| LV ejection fraction | [%] | 53.44±7.45 | 44.51±9.07 | **0.002**^T^ |
| LV ejection fraction < 35% | [n, %] | 0(0%) | 1(6.3%) | 0.400^F^ |
| LA area | [cm2] | 19.17±3.65 | 19.13±3.89 | 0.977^T^ |
| LA volume index | [mL/m^2^] | 35.00±12.09 | 29.32±10.43 | 0.139^T^ |
| LA ejection fraction | [%] | 37.62±6.8 | 32.33±11.07 | 0.088^T^ |
| LV mass index | [g/m^2^] | 63.95±10.63 | 74.26±19.16 | **0.036**^T^ |
| LV end-diastolic volume | [mL] | 158.05±34.58 | 190.26±49.74 | **0.022**^T^ |
| ACE-inhibitor/angiotensin-receptor blocker | [n, %] | 20(90.9%) | 15(100%) | 0.631^F^ |
| β-blocker | [n, %] | 19(86.3%) | 14(93.3%) | 0.681^F^ |
| Loop diuretics | [n, %] | 8(38%) | 6(42.8%) | 0.778^Chi^ |
| Mineralocorticoid receptor antagonists | [n, %] | 6(27.2%) | 5(35.7%) | 0.728^F^ |
| **Legend:** BNP: brain natriuretic peptide; EF: ejection fraction; eGFR: estimated glomerular filtration rate; LA: left atrium; LV: left ventricle; SD: standard deviation; SDB: sleep-disordered breathing; STEMI: ST-elevation myocardial infarction.  Bold values mean statistical significance calculated by the two-sided Student‘s t-test (T), Chi-Square-test (Chi), or Fischer‘s exact test (F). ¶-Data at baseline | | | | |
